# Tissue Distribution and Elimination of Isavuconazole following Single and Repeat Oral-Dose Administration of Isavuconazonium Sulfate to Rats

**DOI:** 10.1128/AAC.01292-17

**Published:** 2017-11-22

**Authors:** Anne-Hortense Schmitt-Hoffmann, Kota Kato, Robert Townsend, Michael J. Potchoiba, William W. Hope, David Andes, Jochen Spickermann, Marlowe J. Schneidkraut

**Affiliations:** a Basilea Pharmaceutica International Ltd., Basel, Switzerland; b Analysis & Pharmacokinetics Research Laboratories, Astellas Pharma Inc., Osaka, Japan; c Astellas Pharma Global Development, Inc., Northbrook, Illinois, USA; d Covance Laboratories, Inc., Madison, Wisconsin, USA; e University of Liverpool, Liverpool, United Kingdom; f University of Wisconsin, Madison, Wisconsin, USA; g Astellas Research Institute of America, Northbrook, Illinois, USA

**Keywords:** isavuconazole, isavuconazonium sulfate, quantitative whole-body autoradiography, rat, tissue distribution, tissue penetration

## Abstract

Quantitative whole-body autoradiography was used to assess the distribution and tissue penetration of isavuconazole in rats following single and repeated oral-dose administration of radiolabeled isavuconazonium sulfate, the prodrug of isavuconazole. Following a single-dose administration of radiolabeled isavuconazonium sulfate (labeled on the active moiety), radioactivity was detectable within 1 h postdose in 56 of 65 tissue/fluid specimens. The highest maximum concentrations (*C*_max_) were observed in bile and liver (66.6 and 24.7 μg eq/g, respectively). The lowest *C*_max_ values were in bone and eye lens (0.070 and 0.077 μg eq/g, respectively). By 144 h postdose, radioactivity was undetectable in all tissues/fluids except liver (undetectable at 336 h) and adrenal gland tissues (undetectable at 672 h). Following daily administration for up to 21 days, 1-h-postdose *C*_max_ values were the highest on or before day 14 in all except seven tissues/fluids, of which only rectum mucosa and small intestine mucosa had *C*_max_ values >25% higher than all other 1-h-postdose values. For 24-h-postdose *C*_max_ values, only large intestine, large intestine mucosa, and urine had the highest *C*_max_ values at day 21. The penetration of single oral doses of unlabeled isavuconazole (25 mg/kg of body weight isavuconazonium sulfate) and voriconazole (50 mg/kg) into rat brain (assessed using liquid chromatography-tandem mass spectrometry) was also compared. Brain concentration/plasma concentration ratios reached approximately 1.8:1 and 2:1, respectively. These data suggest that isavuconazole penetrates most tissues rapidly, reaches a steady state in most or all tissues/fluids within 14 days, does not accumulate in tissues/fluids over time, and achieves potentially efficacious concentrations in the brain.

## INTRODUCTION

Invasive fungal infections (IFIs), such as invasive aspergillosis (IA), present a considerable therapeutic challenge, particularly among immunocompromised patient populations, such as hematopoietic stem cell transplant recipients (1) or those with hematological malignancies (2). The tissue penetration of a systemically administered antifungal drug is a key aspect affecting efficacy (3). The concentration-time course and the absolute concentration obtained in tissues may be markedly different from those obtained in the bloodstream (3, 4). Pharmacological factors (drug formulations, route of administration), physiological factors (inflammation, underlying disease, the blood-brain barrier), and lipophilicity are determinants of the penetration of drugs in tissues (3). Considerable variability in the relative distribution of antifungal agents in plasma and various tissues has been observed. For example, fluconazole concentrations in brain tissue have been shown to be similar to those in plasma in a study in patients with cerebral tumors (5), while the amphotericin B distribution in autopsy samples was variable, with higher levels of accumulation in liver and spleen than brain (6). Caspofungin also showed a variable distribution in the tissues of healthy men, with the highest concentrations being reported in liver, kidney, lung, and spleen but little being detected in the brain or the eye (7). In a study of human autopsy samples, voriconazole effectively penetrated all subcompartments within the central nervous system (CNS) (8).

Isavuconazonium sulfate, the prodrug of the active triazole antifungal drug isavuconazole, is available in both oral and intravenous (i.v.) formulations. Following administration, isavuconazonium sulfate is rapidly cleaved to release the active isavuconazole moiety and an inactive moiety, BAL8728 (9, 10). Isavuconazole has broad-spectrum activity against molds, yeasts, and dimorphic fungi (11–13). *In vivo* antifungal activity has been shown in animal models of invasive systemic and pulmonary aspergillosis as well as mucormycosis (9, 14–19). In these murine models of disseminated fungal infections, isavuconazole treatment reduced the fungal burden in the lung, liver, kidney, and brain, suggesting that isavuconazole achieved effective concentrations at these sites. Phase 3 studies in patients demonstrated the efficacy and safety of isavuconazole for the treatment of IA (20) and mucormycosis (21). As a result, isavuconazonium sulfate is approved by the Food and Drug Administration (FDA) for the treatment of IA and mucormycosis in adults and by the European Medicines Agency for the treatment of IA in adults and for the treatment of mucormycosis in adults when amphotericin B is inappropriate.

Herein we report the results of studies conducted using radiolabeled isavuconazonium sulfate to determine the tissue penetration and distribution of isavuconazole drug-derived radioactivity in various tissues of rats following single or repeated oral-dose administration in rats using quantitative whole-body autoradiography (QWBA). In addition, the penetration of isavuconazole in rat brain was compared with that of voriconazole following a single oral dose of either drug.

## RESULTS

### QWBA studies of ^14^C-labeled isavuconazonium sulfate in pigmented rats following single oral-dose administration.

**FIG 1:**
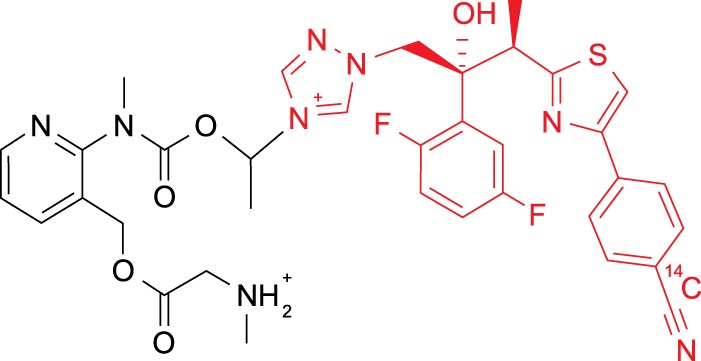
Structure of [cyano-^14^C]isavuconazonium sulfate. Red, isavuconazole; black, inactive prodrug moiety (BAL8728); ^14^C, the position of the radiolabel.

**FIG 2:**
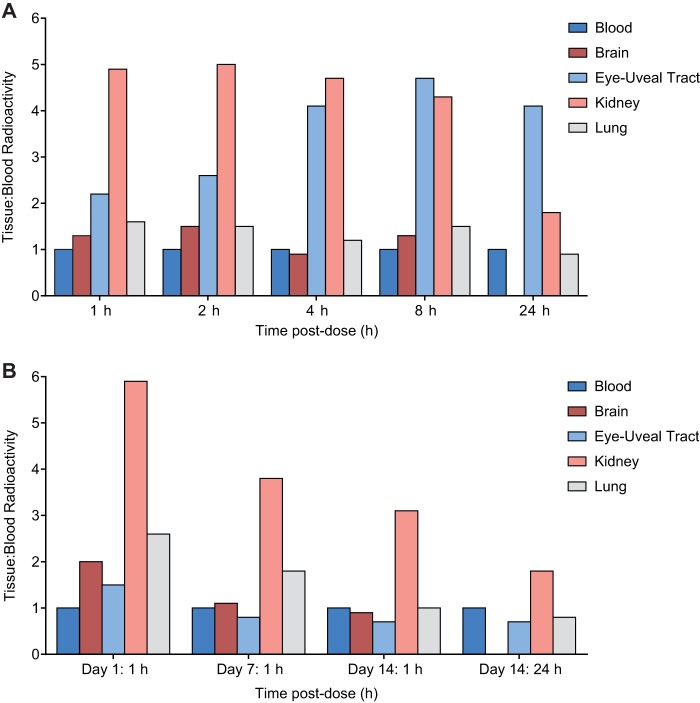
Tissue concentration/blood concentration ratios of ^14^C radioactivity determined by quantitative whole-body autoradiography (QWBA) at specified time points after single oral administration (A) or repeated oral administration (B) of [cyano-^14^C]isavuconazonium sulfate to male rats.

To assess the tissue penetration and distribution of isavuconazole with single oral doses of isavuconazonium sulfate, rats were given ^14^C-labeled isavuconazonium sulfate orally at 5 mg/kg of body weight, with labeling being on the active moiety (Fig. 1), prior to sacrifice between 1 and 672 h later (see Materials and Methods). Radioactivity was quantifiable in most tissues and fluids by 1 h postdose and reached a maximum within 2 h postdose in blood, other fluids, and most tissues except the eye lens and fat tissues (mesenteric, reproductive, and white fat; all at 8 h) (Table 1). At assessment times of <24 h postdose, most tissue concentrations exceeded those in blood, indicating partitioning of drug-derived radioactivity into most tissues. High levels of radioactivity were found in the kidneys and the uveal tract up to 8 h postdose, and tissue concentration/blood concentration ratios frequently exceeded 2:1 (Fig. 2A). The highest maximum concentration (*C*_max_) values were observed in bile, liver, adrenal gland, adrenal cortex, and brown fat (66.6 [2 h postdose], 24.7 [1 h postdose], 10.6 [2 h postdose], 11.0 [2 h postdose], and 3.76 [2 h postdose] μg eq/g, respectively). The lowest *C*_max_ values were in bone, eye lens, eye, seminal vesicles, olfactory bulb, and blood (0.070, 0.077, 0.312, 0.341, 0.591, and 0.603 μg eq/g, respectively). By 72 h postdose, the amount of radioactivity in blood and all tissues and fluids declined below measurable levels, except as follows: nasal mucosa, preputial gland, pigmented skin, stomach, stomach mucosa, testis, urine, and uveal tract (all undetectable by 144 h); liver (undetectable by 336 h); and adrenal glands, including the cortex and medulla (undetectable by 672 h). The concentrations of drug-derived radioactivity were similar and decreased with the same pattern in pigmented and nonpigmented skin, indicating no additional affinity for melanin.

**TABLE 1:** Concentrations of radioactivity in tissues and fluids determined by QWBA at specified times after single oral-dose administration of [cyano-^14^C]isavuconazonium sulfate in male pigmented rats^*c*^

Matrix	Concn of [cyano-^14^C]isavuconazonium sulfate (μg eq/g) at:								
	1 h	2 h	4 h	8 h	24 h	72 h	144 h	336 h	672 h
Adrenal cortex	5.76^*a*^	11.0^*a*^	7.14	7.91	2.46	1.03	0.348	0.169	BLQ^*a*^
Adrenal gland(s)	5.05^*a*^	10.6^*a*^	6.99	7.50	2.31	0.908	0.270	0.136	BLQ^*a*^
Adrenal medulla	2.58^*a*^	4.44^*a*^	5.05	4.01	1.32	0.317	0.085	0.047	BLQ^*a*^
Aorta	0.640	1.14	0.339	0.427	ND	ND	ND	ND	ND
Arterial wall	0.645	0.923	0.348	0.465	ND	ND	ND	ND	ND
Bile	147	66.6	21.6	19.0	ND	ND	ND	ND	ND
Blood	0.463	0.603	0.252	0.247	0.092	BLQ	ND	ND	ND
Bone	0.048	0.070	BLQ	BLQ	BLQ	ND	ND	ND	ND
Bone marrow	0.717	0.822	0.287	0.279	0.054	ND	ND	ND	ND
Brain cerebellum	0.597	0.887	0.234	0.322	BLQ	ND	ND	ND	ND
Brain cerebrum	0.533	0.808	0.216	0.272	BLQ	ND	ND	ND	ND
Brain medulla	0.641	0.912	0.249	0.321	BLQ	ND	ND	ND	ND
Brain olfactory lobe	0.485	0.591	0.189	0.234	BLQ	ND	ND	ND	ND
Bulbourethral gland	0.846	1.54	0.543	0.534	0.094	ND	ND	ND	ND
Cecum	1.47	0.725	0.696	0.625	0.448	ND	ND	ND	ND
Cecum mucosa	1.33	1.01	1.01	0.916	0.512	ND	ND	ND	ND
Diaphragm	0.879	1.25	0.385	0.501	0.079	ND	ND	ND	ND
Epididymis	0.556	1.42^*a*^	0.754	0.577	0.116	BLQ	ND	ND	ND
Esophagus	0.721	0.974	0.346	0.382	0.081	BLQ	ND	ND	ND
Exorbital lacrimal gland	1.35	1.94	0.675	0.698	0.106	BLQ	ND	ND	ND
Eye lens	BLQ	0.050	BLQ	0.077	0.060	ND	ND	ND	ND
Eye uveal tract	1.02	1.59	1.04	1.15	0.374	0.097	ND	ND	ND
Eye(s)	0.180	0.312	0.200	0.191	0.084	BLQ	ND	ND	ND
Fat (brown)	1.27	3.76	1.35	1.26	0.142	BLQ	ND	ND	ND
Fat (mesenteric)	0.784	2.34	2.68	3.24	0.287	BLQ	ND	ND	ND
Fat (reproductive)	0.557	1.84	2.11	2.79	0.747	BLQ	ND	ND	ND
Fat (white)	1.04	2.47	2.50	2.84	0.275	BLQ	ND	ND	ND
Harderian gland	1.66	3.74	1.13	1.11	0.165	ND	ND	ND	ND
Intra-orbital lacrimal gland	1.27	1.91	0.675	0.675	0.129	ND	ND	ND	ND
Kidney cortex	2.04	2.77	1.14	1.12	0.176	BLQ	ND	ND	ND
Kidney medulla	2.40	3.29	1.21	0.998	0.159	BLQ	ND	ND	ND
Kidney(s)	2.29	3.04	1.19	1.06	0.170	BLQ	ND	ND	ND
Large intestine	0.789	1.05	0.402	0.650	0.206	BLQ	ND	ND	ND
Large intestine mucosa	0.879	1.11	0.470	0.781	0.312	BLQ	ND	ND	ND
Liver	24.7	15.7	6.21	5.31	1.45	0.392	0.138	BLQ	ND
Lung(s)	0.743	0.915	0.292	0.381	0.082	BLQ	ND	ND	ND
Lymph node(s)	0.529	0.651	0.289	0.300	0.054	ND	ND	ND	ND
Lymph node(s), mandibular	0.577	0.730	0.263	0.317	0.048	ND	ND	ND	ND
Muscle	0.530	0.812	0.255	0.278	BLQ	BLQ	ND	ND	ND
Myocardium	1.03	1.47	0.489	0.589	0.080	BLQ	ND	ND	ND
Nasal mucosa	0.589	1.38	0.467	0.436	0.124	0.042	ND	ND	ND
Pancreas	1.49	1.60	0.676	0.769	0.080	BLQ	ND	ND	ND
Pineal body	NR	1.13	0.345	0.490	ND	ND	ND	ND	ND
Pituitary gland	0.811	1.25	0.431	0.535	0.084	ND	ND	ND	ND
Preputial gland	0.576^*a*^	1.47^*a*^	0.900^*a*^	1.28^*a*^	0.202^*a*^	0.073	ND	ND	ND
Prostate gland	0.975	1.71	0.608	0.572	0.069	BLQ	ND	ND	ND
Rectum mucosa	0.581	1.04	0.207	0.513	0.221	BLQ	ND	ND	ND
Salivary gland(s)	1.12	1.52	0.485	0.531	0.070	ND	ND	ND	ND
Seminal vesicle(s)	0.157	0.341	0.194	0.163	0.059	BLQ	ND	ND	ND
Skin (nonpigmented)	0.378	0.872	0.325	0.321	0.080	BLQ	ND	ND	ND
Skin (pigmented)	0.520	0.910	0.511	0.461	0.121	0.052	ND	ND	ND
Small intestine	1.50	1.72	1.07	0.970	0.259	ND	ND	ND	ND
Small intestine mucosa	2.52	2.18	1.78	1.45	0.553	ND	ND	ND	ND
Spinal cord	0.595	0.948	0.284	0.315	BLQ	ND	ND	ND	ND
Spleen	0.763	0.850	0.306	0.380	0.093	BLQ	ND	ND	ND
Stomach	1.21	1.77	0.540	0.513	0.093	0.077	ND	ND	ND
Stomach mucosa	2.22	2.43	0.891	0.614	0.104	0.103	ND	ND	ND
Stomach wall	0.946	1.01	0.356	0.362	0.086	BLQ	ND	ND	ND
Testis(es)	0.507	1.03	0.520	0.512	0.146	0.070	ND	ND	ND
Thymus	0.543	0.827	0.259	0.287	0.072	ND	ND	ND	ND
Thyroid	0.858	1.09	0.389	0.447	0.104	ND	ND	ND	ND
Tongue	0.954	1.17	0.428	0.422	0.049	ND	ND	ND	ND
Tooth pulp	0.592	0.628	0.261	0.216	0.048	ND	ND	ND	ND
Urinary bladder	ND^*b*^	1.63^*a*^	0.535	0.406	ND	ND	ND	ND	ND
Urine	17.5	54.7	8.61	8.95	2.90	0.195	ND	ND	ND

a The tissue appeared to be fat soaked.

b The urinary bladder could not be sampled due to the flare from the urine.

c [cyano-^14^C]isavuconazonium sulfate was administered at 5 mg/kg. The correction factor of 1.1352 (sulfate/free form) was applied to calculate the concentrations of free base equivalents of radioactivity in tissues, bile, blood, and urine. BLQ, below the limit of quantitation (<0.0414 μg equivalents [cyano-^14^C]isavuconazonium sulfate/g); ND, not detectable (the borders of the sample were indiscernible from the background or surrounding tissue); NR, not represented (tissue was not present in the section); QWBA, quantitative whole-body autoradiography.

The amount of radioactivity in whole blood and plasma measured by liquid scintillation counting (LSC) was consistent with that measured by QWBA. The *C*_max_ for both blood and plasma radioactivity occurred at 2 h postdose at 0.603 and 0.891 μg eq/g, respectively, resulting in a ratio of 0.620 at the time of *C*_max_ (*T*_max_). Blood concentration/plasma concentration ratios were 0.620 to 0.782 through 24 h postdose but increased to 1.06 and 2.38 at 72 h and 144 h, respectively, indicating either a slow redistribution from plasma to blood cells through 24 h or a slower clearance from blood cells than plasma at later times. The elimination half-lives in blood and plasma were 340 h and 28.1 h, respectively. The area under the concentration-time curve from time zero to infinity (AUC_inf_) for blood and plasma were 17.3 μg eq · h/ml and 13.4 μg eq · h/ml, respectively.

### QWBA study of ^14^C-labeled isavuconazonium sulfate in albino rats following repeated oral-dose administration.

To assess the distribution and potential accumulation of isavuconazole following repeated oral doses of isavuconazonium sulfate, rats were given ^14^C-labeled isavuconazonium sulfate at 30 mg/kg daily for up to 21 days and sacrificed at times between 1 h and 672 h postdose (see Materials and Methods). In 31 of 62 tissue and fluid specimens for which 1-h-postdose samples on day 1 were available for comparison, the concentrations of radioactivity were similar (<2-fold increase) at 1 h postdose on days 7, 14, and 21 (Tables 2 and 3). Among the tissues and fluids for which ≥2-fold increases for *C*_max_ values were seen in 1-h-postdose samples subsequent to day 1, *C*_max_ was observed on day 7 in 4 tissues and fluids (bile, reproductive fat, seminal vesicles, and stomach wall), on day 14 in 20 tissues and fluids (adrenal tissues [adrenal gland, cortex, medulla], intestinal tissues [cecum, cecum mucosa, large intestine, large intestine mucosa], reproductive tissues [bulbourethral gland, epididymis, testes], lymph tissues [lymph nodes, mandibular lymph nodes], aorta, blood, intraorbital lacrimal gland, kidney medulla, nasal mucosa, spinal cord, tooth pulp, and urine), and on day 21 in 7 tissues and fluids (intestinal tissues [rectum mucosa, small intestine, small intestine mucosa], mesenteric fat, pineal body, nonpigmented skin, and thyroid). Among tissues and fluids with a 1-h-postdose *C*_max_ on day 21, only two tissues, rectum mucosa and small intestine mucosa, had concentrations that were >25% higher than those at 1 h postdose on any previous assessment day. These higher levels were possibly due to contamination by residual intestinal contents. These data suggest that steady-state concentrations were achieved in most or all tissues and fluids on or before day 14.

**TABLE 2:** Concentrations of radioactivity in tissues and fluids determined by QWBA at specified times after oral administration of [cyano-^14^C]isavuconazonium sulfate for up to 21 consecutive days in male albino rats^*b*^

Matrix	Concn of [cyano-^14^C]isavuconazonium sulfate (μg eq/g) at the indicated times after the last dose on:							
	Day 1				Day 7		Day 14	
	1 h	4 h	8 h	24 h	1 h	24 h	1 h	24 h
Adrenal cortex	10.1^*a*^	24.1^*a*^	19.0^*a*^	6.54^*a*^	20.9^*a*^	14.3^*a*^	23.1^*a*^	16.9^*a*^
Adrenal gland(s)	9.51^*a*^	22.7^*a*^	17.1^*a*^	5.83^*a*^	19.3^*a*^	12.4^*a*^	21.2^*a*^	14.0^*a*^
Adrenal medulla	8.04^*a*^	17.9^*a*^	10.8^*a*^	3.47^*a*^	11.7^*a*^	6.37^*a*^	19.2^*a*^	5.12^*a*^
Aorta	2.00	4.67	3.76	ND	2.75	1.51	4.53	2.35
Arterial wall	ND	5.53	ND	ND	1.88	ND	3.43	ND
Bile	101	198	109	27.3	472	27.7	157	ND
Blood	0.783	1.99	1.81	ND	1.70	1.34	3.04	1.41
Bone	BLQ	BLQ	BLQ	ND	BLQ	BLQ	BLQ	BLQ
Bone marrow	2.35	4.23	3.41	ND	1.90	0.979	4.03	BLQ
Brain cerebellum	1.55	3.28	2.11	ND	1.81	BLQ	2.74	BLQ
Brain cerebrum	1.51	3.36	2.02	ND	1.86	BLQ	2.72	BLQ
Brain medulla	1.60	3.52	2.22	ND	1.79	BLQ	2.93	BLQ
Brain olfactory lobe	1.46	2.93	1.70	ND	1.44	BLQ	2.46	BLQ
Bulbourethral gland	2.21	6.14	4.67	ND	3.08	0.933	4.60	1.27
Cecum	BLQ	12.9	9.22	7.13	6.41	6.57	13.5	13.6
Cecum mucosa	4.12	15.8	13.7	7.42	16.6	11.4	18.2	10.9
Diaphragm	2.86	5.22	3.97	BLQ	3.52	0.920	5.01	1.27
Epididymis	1.00^*a*^	4.88	3.07	ND	1.57	0.819^*a*^	2.26^*a*^	0.925^*a*^
Esophagus	3.00	4.44	2.51	0.896	2.10	1.19	2.89	BLQ
Exorbital lacrimal gland	3.62	9.17	7.04	0.881	3.94	1.37	6.64	1.21
Eye lens	BLQ	BLQ	BLQ	ND	BLQ	BLQ	BLQ	BLQ
Eye uveal tract	1.21	2.40	1.31	ND	1.39	1.06	2.16	0.948
Eye(s)	BLQ	BLQ	BLQ	ND	BLQ	BLQ	0.787	BLQ
Fat (brown)	5.34	8.80	8.56	0.939	7.00	1.69	9.92	2.20
Fat (mesenteric)	0.802	23.4	21.0	1.80	2.96	2.21	1.17	2.67
Fat (reproductive)	BLQ	16.0	15.5	2.27	3.76	3.66	2.03	3.73
Fat (white)	1.74	26.7	16.8	1.59	2.53	2.20	2.39	1.42
Harderian gland	3.63	12.6	9.14	1.03	4.55	1.73	6.58	1.38
Intra-orbital lacrimal gland	3.34	7.94	6.51	ND	3.38	1.39	6.77	1.33
Kidney cortex	4.67	10.4	6.96	ND	6.60	2.42	9.24	2.73
Kidney medulla	4.64	9.76	6.43	ND	6.10	2.43	9.31	2.33
Kidney(s)	4.65	10.2	6.80	1.42	6.46	2.43	9.29	2.58
Large intestine	BLQ	3.25	4.05	1.91	3.49	1.90	4.00	1.46
Large intestine mucosa	2.55	4.80	2.88	1.92	3.21	4.06	5.32	2.31
Liver	35.8	38.3	31.5	7.53	40.1	15.6	58.8	18.4
Lung(s)	2.03	4.61	3.49	0.757	3.13	1.41	3.14	1.16
Lymph node(s)	1.22	3.57	3.02	ND	2.06	0.987	2.80	1.12
Lymph node(s), mandibular	1.35	3.61	2.78	ND	1.82	ND	2.96	ND
Muscle	1.32	3.64	2.43	BLQ	1.59	BLQ	1.84	BLQ
Myocardium	3.10	6.79	4.32	0.964	3.99	1.49	5.60	1.83
Nasal mucosa	1.36	6.08	3.64	0.895	1.98	1.19	3.17	1.76
Pancreas	3.99	8.09	5.77	0.749	4.20	1.05	6.34	1.17
Pineal body	1.84	5.31	3.93	ND	4.13	ND	4.47	ND
Pituitary gland	2.50	4.61	2.80	ND	ND	ND	4.85	ND
Preputial gland	ND	8.32^*a*^	12.0^*a*^	ND	4.00^*a*^	3.12^*a*^	4.52^*a*^	2.20^*a*^
Prostate gland	2.78	13.0	5.39	BLQ	3.24	1.10	3.23	1.17
Rectum mucosa	0.989	2.61	2.00	0.821	1.75	0.778	1.55	1.30
Salivary gland(s)	2.91	5.91	4.29	BLQ	3.56	0.868	5.03	0.941
Seminal vesicle(s)	BLQ	3.32	1.62	BLQ	1.97	0.783	1.63	0.938
Skin (nonpigmented)	1.06	3.64	3.33	BLQ	1.87	0.988	2.16	1.01
Small intestine	1.74	4.50	12.9	3.65	9.95	3.07	10.0	2.57
Small intestine mucosa	3.15	14.6	21.7	7.81	16.7	7.26	21.9	9.43
Spinal cord	1.41	3.74	2.16	ND	1.80	ND	2.92	BLQ
Spleen	2.12	3.68	3.28	ND	2.47	1.00	4.02	1.11
Stomach	4.31	5.10	4.41	BLQ	4.74	1.56	5.93	1.11
Stomach mucosa	5.48	7.97	6.16	ND	6.27	ND	9.00	ND
Stomach wall	1.57	2.61	1.98	ND	3.19	ND	2.43	ND
Testis(es)	1.01	3.21	2.28	BLQ	1.50	0.882	2.26	1.00
Thymus	1.42	2.86	2.03	BLQ	1.53	BLQ	2.26	BLQ
Thyroid	2.15	5.39	3.76	ND	4.88	2.08	5.23	2.52
Tongue	2.43	5.70	3.59	BLQ	3.22	0.927	4.27	0.970
Tooth pulp	1.23	2.50	1.71	ND	1.67	1.07	3.07	0.884
Urinary bladder	5.39	9.97	3.69	3.77	4.79	1.71	2.67	12.8
Urine	8.85	119	102	9.75	16.6	16.4	58.0	20.4

a Tissue appeared to be fat soaked.

b [cyano-^14^C]isavuconazonium sulfate was administered at 30 mg/kg/day. BLQ, below the limit of quantitation (the lower limit of quantitation for day 1 was <0.742 μg eq [cyano-^14^C]isavuconazonium sulfate/g; the lower limit of quantitation for days 7 and 14 was <0.748 μg eq [cyano-^14^C]isavuconazonium sulfate/g); ND, not detectable (the borders of the sample were not discernible from the background or surrounding tissue); QWBA, quantitative whole-body autoradiography.

**TABLE 3:** Concentrations of radioactivity in tissues and fluids determined by QWBA at specified times after oral administration of [cyano-^14^C]isavuconazonium sulfate for 21 consecutive days in male albino rats^*e*^

Matrix	Concn of [cyano-^14^C]isavuconazonium sulfate (μg eq/g) at:								
	Predose^*a*^	1 h^*a*^	4 h^*a*^	8 h^*a*^	24 h^*a*^	72 h^*a*^^,^^*b*^	168 h^*a*^	336 h^*a*^	672 h^*a*^
Adrenal cortex	17.2^*c*^	22.8^*c*^	33.6^*c*^	26.4^*c*^	12.7^*c*^	10.4	4.45^*c*^	2.43^*c*^	1.29
Adrenal gland(s)	14.3^*c*^	20.7^*c*^	29.2^*c*^	22.4^*c*^	11.4^*c*^	7.78	3.81^*c*^	2.14^*c*^	1.13
Adrenal medulla	6.86^*c*^	8.12^*c*^	13.3^*c*^	10.5^*c*^	7.21^*c*^	3.41	1.94^*c*^	1.02^*c*^	BLQ
Aorta	1.20	4.22	6.37	3.44	ND	ND	ND	ND	ND
Arterial wall	ND	ND	6.91	3.12	ND	ND	ND	ND	ND
Bile	37.6	186	231	194	25.3	ND	ND	ND	ND
Blood	1.21	2.60	3.73	2.18	1.01	BLQ	BLQ	BLQ	ND
Bone	BLQ	BLQ	BLQ	BLQ	BLQ	ND	ND	ND	ND
Bone marrow	0.975	3.24	7.10	2.34	BLQ	ND	ND	ND	ND
Brain cerebellum	BLQ	2.59	5.15	1.70	BLQ	ND	ND	ND	ND
Brain cerebrum	BLQ	2.66	5.45	1.68	BLQ	ND	ND	ND	ND
Brain medulla	0.813	2.79	5.45	1.88	BLQ	ND	ND	ND	ND
Brain olfactory lobe	BLQ	2.34	4.52	1.69	BLQ	ND	ND	ND	ND
Bulbourethral gland	1.24	4.27	6.78	3.36	0.788	ND	ND	ND	ND
Cecum	8.39	9.97	24.0	21.2	ND	1.08	BLQ	ND	ND
Cecum mucosa	20.1	16.6	43.7	40.5	ND	ND	ND	ND	ND
Diaphragm	1.13	3.84	7.51	3.09	0.753	0.764	BLQ	ND	ND
Epididymis	0.998^*c*^	2.22	5.93^c^	3.09^c^	BLQ^*c*^	BLQ	BLQ	ND	ND
Esophagus	0.928	2.52	4.29	2.59	BLQ	BLQ	ND	ND	ND
Exorbital lacrimal gland	1.26	5.60	11.2	5.04	1.20	BLQ	BLQ	BLQ	ND
Eye lens	BLQ	0.794	1.35	0.830	BLQ	ND	ND	ND	ND
Eye uveal tract	BLQ	2.22	4.58	2.20	1.17	ND	ND	ND	ND
Eye(s)	BLQ	BLQ	1.28	0.775	BLQ	ND	ND	ND	ND
Fat (brown)	2.30	9.33	17.7	8.62	1.50	1.19	1.11	BLQ	ND
Fat (mesenteric)	1.46	3.43	18.7	7.62	2.33	0.781	BLQ	ND	ND
Fat (reproductive)	3.59	3.62	10.9	8.01	2.99	BLQ	BLQ	BLQ	ND
Fat (white)	1.46	2.75	12.1	7.97	1.04	0.830	BLQ	ND	ND
Harderian gland	1.50	6.58	18.9	7.23	1.06	ND	ND	ND	ND
Intra-orbital lacrimal gland	1.39	5.89	11.0	5.36	1.32	ND	ND	ND	ND
Kidney cortex	2.64	8.76	16.1	7.53	2.33	1.64	1.05	0.969	BLQ
Kidney medulla	2.69	8.49	12.3	7.02	1.97	1.29	BLQ	BLQ	BLQ
Kidney(s)	2.64	8.59	13.7	7.33	2.16	1.52	0.871	0.885	BLQ
Large intestine	6.87	3.65	6.41	4.44	1.92	BLQ	BLQ	ND	ND
Large intestine mucosa	ND^*d*^	4.37	9.18	4.99	5.15	ND	ND	ND	ND
Liver	14.6	54.4	64.4	41.4	14.2	7.80	2.95	1.09	BLQ
Lung(s)	0.957	3.38	6.34	3.70	0.814	BLQ	BLQ	ND	ND
Lymph node(s)	BLQ	2.44	5.54	2.70	ND	ND	ND	ND	ND
Lymph node(s), mandibular	BLQ	2.87	5.33	2.73	ND	ND	ND	ND	ND
Muscle	BLQ	1.27	4.38	1.71	BLQ	BLQ	ND	BLQ	ND
Myocardium	1.92	5.77	10.7	4.41	1.47	1.36	0.901	0.777	ND
Nasal mucosa	1.87	3.04	6.38	4.03	0.931	0.834	ND	ND	ND
Pancreas	1.05	5.91	10.5	4.10	BLQ	BLQ	BLQ	BLQ	ND
Pineal body	ND	5.49	9.42	3.94	ND	ND	ND	ND	ND
Pituitary gland	ND	2.88	7.46	2.97	ND	ND	ND	ND	ND
Preputial gland	2.29^*c*^	4.85^*c*^	10.8^*c*^	6.68^*c*^	4.65^*c*^	2.02	1.42	ND	ND
Prostate gland	0.998	4.33	13.9	3.66	0.839	BLQ	BLQ	ND	ND
Rectum mucosa	0.886	4.54	4.57	4.63	BLQ	ND	ND	ND	ND
Salivary gland(s)	1.40	4.52	9.15	3.36	BLQ	BLQ	BLQ	ND	ND
Seminal vesicle(s)	BLQ	1.92^*c*^	2.89	1.64	BLQ	BLQ	BLQ	ND	ND
Skin (nonpigmented)	1.01	2.25	3.85	2.68	BLQ	BLQ	BLQ	BLQ	ND
Small intestine	4.59	12.1	18.9	11.3	2.38	ND	ND	ND	ND
Small intestine mucosa	7.89	32.5	39.6	26.9	13.6	ND	ND	ND	ND
Spinal cord	BLQ	2.68	5.84	1.76	ND	ND	ND	ND	ND
Spleen	1.40	3.50	6.76	3.17	1.09	0.830	BLQ	BLQ	BLQ
Stomach	1.58	3.61	7.41	3.93	1.14	ND	ND	BLQ	ND
Stomach mucosa	ND	7.38	14.0	4.65	ND	ND	ND	ND	ND
Stomach wall	ND	2.27	3.88	2.13	ND	ND	ND	ND	ND
Testis(es)	0.925	2.21	5.03	2.15	0.752	BLQ	BLQ	BLQ	ND
Thymus	BLQ	2.43	4.64	1.90	BLQ	BLQ	BLQ	BLQ	ND
Thyroid	2.09	5.74	10.6	4.05	3.23	2.20	2.78	1.56	ND
Tongue	1.15	4.04	8.00	3.10	0.809	ND	ND	ND	ND
Tooth pulp	1.23	2.79	4.30	3.48	ND	ND	ND	ND	ND
Urinary bladder	1.09	3.60	ND^*d*^	19.0	ND	ND	ND	ND	ND
Urine	9.28	21.5	148	67.9	24.0	1.58	BLQ	ND	ND

a The last dose that these animals received was on day 21.

b The actual collection time point was 73.4 h postdose, as the sample was collected approximately 88 min after the scheduled collection time (approximately 28 min outside of the acceptable standard range).

c The tissue appeared to be fat soaked.

d Not detectable due to flare from gastrointestinal contents (large intestine mucosa) or urine (urinary bladder).

e [cyano-^14^C]isavuconazonium sulfate was administered at 30 mg/kg/day. Dose times are relative to the day 21 dose. BLQ, below the limit of quantitation (the lower limit of quantitation for day 21 was <0.748 μg eq [cyano-^14^C]isavuconazonium sulfate/g); ND, not detectable (the sample shape was not discernible from the background or surrounding tissue); QWBA, quantitative whole-body autoradiography.

In 24-h-postdose samples from days 1, 7, 14, and 21, radioactivity was frequently undetectable (Tables 2 and 3). Among 27 tissues and fluids for which day 1 samples were available for comparison, <2-fold increases were observed at any subsequent assessment day in 17 tissues. Tissues with the highest 24-h-postdose *C*_max_ on day 14 included adrenal tissues (adrenal glands, adrenal cortex), cecum mucosa, brown fat, liver, and urinary bladder. The tissues and fluids with the highest 24-h-postdose *C*_max_ on day 21 included large intestine tissues (large intestine, large intestine mucosa) and urine. These data suggest that there was no substantial accumulation of isavuconazole in any tissues.

The tissue/fluid distribution patterns and the elimination of radioactivity from most tissues and fluids in albino rats that received repeated oral-dose administration of radiolabeled isavuconazonium sulfate were largely similar through the 24-h-postdose observation period to those in the same strain of rats administered a single dose of radiolabeled isavuconazonium sulfate. Adrenal gland tissues and the liver consistently had among the highest *C*_max_ values at all time points, whereas bone, eye lens, and eye consistently had among the lowest *C*_max_ values at all time points. In LSC analyses, day 21 blood and plasma elimination half-lives were 319 and 48.2 h, respectively. The blood and plasma AUC_inf_ values on day 1 were 67.6 and 105 μg eq · h/ml, respectively. The elimination half-life of each tissue on day 1 could not be calculated because the sampling time on day 1 was limited to 24 h. The AUC_inf_ values of the free base equivalents of total radioactivity were ∼4-fold higher for blood than plasma (550 and 135 μg eq · h/ml, respectively) after 21 consecutive daily doses.

Similar to the incremental increase in the concentration of drug-derived radioactivity in tissues between days 1 and 14, the concentration of drug-derived radioactivity in blood also increased upon repeated administration. The radioactivity increased more markedly in blood than in tissues, so that overall the tissue concentration/blood concentration ratios decreased between days 1 and 14. The tissue concentration/blood concentration ratios at 1 h postdose on days 1, 7, and 14 and 24 h postdose on day 14 were examined for the same tissues assessed in the single-dose study (Fig. 2B). Similar to the observation for single-dose administration, the highest ratios were observed for kidney, which were >2:1 at the 1-h-postdose time points on each assessment day. The ratios for all tissues decreased decrementally in 1-h-postdose samples from days 7 and 14, reflecting the greater incremental increases in the blood concentrations at those time points. By 24 h postdose on day 14, isavuconazole-associated radioactivity was undetectable in the brain (Fig. 2B).

### Oral dosing of unlabeled isavuconazonium sulfate in rats: brain and plasma levels of isavuconazonium, isavuconazole, and voriconazole.

**FIG 3:**
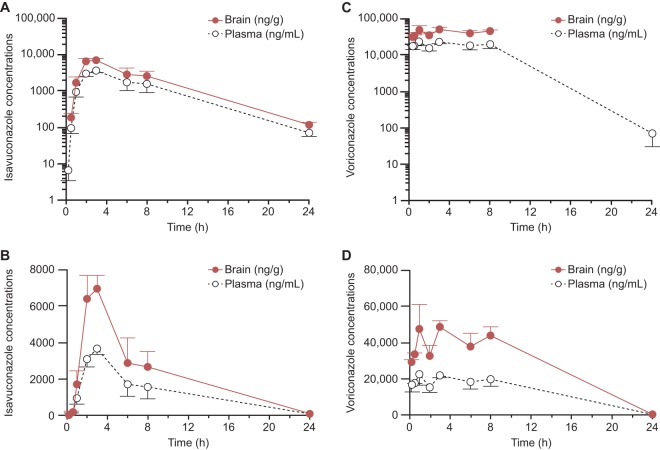
Mean concentrations ± standard deviations of isavuconazole (A, B) and voriconazole (C, D) in rats (*n* = 3 at each time point) following a single oral dose of 25 mg/kg isavuconazole (administered as isavuconazonium sulfate) or voriconazole. (A, C) Log-linear scale; (B, D) linear-linear scale (note the differences in the *y* axis scales).

To further characterize the penetration of isavuconazole in brain and compare it with that of voriconazole, rats were administered a single oral dose of isavuconazole (25 mg/kg) or voriconazole (50 mg/kg), and the concentrations of each drug in brain and plasma samples taken up to 24 h later were assessed (see Materials and Methods). Following an oral dose, the plasma concentration of isavuconazole reached a maximum within 3 h and then declined biphasically (Fig. 3A; Table 4). Isavuconazole concentrations in brain declined in parallel with those in plasma (Fig. 3B; Table 4). The brain concentrations of isavuconazole were approximately twice as high as the plasma concentrations, with an overall mean brain AUC/plasma AUC ratio and mean individual brain AUC/plasma AUC ratios of 1.8:1 (Table 4 and data not shown). The isavuconazonium prodrug was not detectable in plasma or brain (data not shown). The plasma concentrations of voriconazole following an oral dose were more variable, as no clear maximum was observed within 8 h, although as also observed for isavuconazole, the concentrations in brain declined in parallel with those in plasma (Fig. 3C and D). The measurable levels of voriconazole in brain were approximately twice as high as those in plasma (average of the individual brain concentration/plasma concentration ratios, 2:1; data not shown).

**TABLE 4:** Values of pharmacokinetic parameters for isavuconazole and voriconazole in plasma and brain following a single oral administration in rats^*a*^

Compound and matrix	*t*_1/2_ (h)	*T*_max_ (h)	*C*_max_ (μg/ml)	AUC_last_ (μg · h/ml)	*t*_last_ (h)	AUC_inf_ (μg · h/ml)
Isavuconazole						
Brain	3.7	3.0	6.95	53.7	24	54.3
Plasma	3.8	3.0	3.67	30.3	24	30.7
Voriconazole						
Brain	ND	3.0	48.07	321.5	8	ND
Plasma	2.1	1.0	21.97	304.5	24	304.7

a AUC_inf_, area under the concentration-time curve from time zero to infinity; AUC_last_, area under the concentration-time curve from time zero to the last measurable time point; *C*_max_, maximum concentration observed; ND, not determined; *t*_1/2_, half-life; *t*_last_, last measurable time point; *T*_max_, time of maximum concentration observed.

## DISCUSSION

These studies characterized the penetration, distribution, and elimination of isavuconazole with oral dosing of the prodrug isavuconazonium sulfate in rats. Following a single oral dose of radiolabeled prodrug, the maximum concentrations of isavuconazole-associated radioactivity were reached by 2 h in most tissues and fluids and by 8 h in all tissues and fluids. Radioactivity was undetectable after 144 h in almost all tissues except liver (from which drug was eliminated at 336 h) and adrenal gland tissues (from which drug was eliminated at 672 h). With repeated dosing for up to 21 days, steady-state concentrations appeared to be reached by day 14 without strong evidence of accumulation in any tissues. The only tissues or fluids in which substantial increases in 1-h-postdose or 24-h-postdose concentrations were observed after day 14 were intestinal tissues and urine, which might be expected to demonstrate some variability due to potential contamination attributable to oral dosing (intestinal tissues) or variations in the excretion of isavuconazole metabolites (urine). There were no clear differences between radiolabel concentrations in the pigmented and nonpigmented skin, indicating that isavuconazole was unlikely to bind to melanin. Following the administration of single oral doses of unlabeled isavuconazonium sulfate, the brain penetration of isavuconazole was comparable to that of voriconazole, with the concentrations in brain being approximately twice as high as those in plasma. Taken together, these studies suggest that isavuconazole is potentially useful for the treatment of infections with susceptible pathogens in a wide range of tissues, including the CNS.

These observations are consistent with the physicochemical properties of isavuconazole, which include a low molecular weight, a small polar surface area, intermediate lipophilicity, and high permeability (22) and the fact that it is not a substrate for efflux transporters (23). They also provide a basis for the efficacy of isavuconazole seen in models in earlier preclinical studies. For example, in a murine model of disseminated Aspergillus flavus infection using isavuconazole treatment at 24 h postinfection, the clearance of the organism from all organs (lung, liver, kidneys, and brain) was achieved at 14 days postinfection in 5/6, 3/6, and 3/4 survivors receiving 30, 15, and 6 mg/kg dose of the prodrug, respectively (9). The efficacy of isavuconazole for clearance of the tissue burden has been shown not to be specific to aspergillosis, as isavuconazole demonstrated a significant reduction in the fungal brain burden at all doses tested in a murine model of disseminated Candida krusei infection (17). The relevance of these data from preclinical studies for clinical efficacy is also supported by the efficacy of isavuconazole observed in patients with disseminated fungal infections included in the SECURE and VITAL trials (20, 21, 24).

It is well established that the limited brain penetration of many antifungal agents limits their usefulness for the treatment of CNS infections (3). Although voriconazole is not the only agent with efficacy for the treatment of aspergillosis, it has become the standard treatment for CNS aspergillosis because of both its efficacy and its penetration of brain (25, 26). The efficacy of isavuconazole for the treatment of IA has been shown to be comparable to that of voriconazole (20), but the extent of its penetration of the CNS had not previously been assessed. Because isavuconazole is not a substrate for efflux mediated by P-glycoprotein in humans (23), there was reason to believe that isavuconazole might also reach potentially therapeutic concentrations in the brain. As pointed out by others (27), studies of the CNS penetration of other agents have frequently relied on the concentrations observed in cerebrospinal fluid, which may not accurately reflect penetration into tissues. The current study assessed the concentrations in brain tissues directly and observed a 1.8:1 brain concentration/plasma concentration ratio for isavuconazole, similar to that observed for voriconazole (2:1) and to the value reported previously by others for voriconazole in rats (1.88:1) (27). Given that a previous study of voriconazole also found evidence of concentrations in human brain at least 2-fold higher than those in plasma (27), it seems likely that the similarity of the data for both agents in rats will extend to humans as well. The potential utility of isavuconazole for CNS infections is supported by case reports of successful treatment of such infections (28–30). On the other hand, it is unclear whether the much more limited penetration of bone by isavuconazole in rats can be extrapolated to humans and might limit its efficacy to treat those infections.

In addition to any uncertainty over the extent to which the results can be extrapolated to humans, this study has other important limitations. As is the standard practice for QWBA studies, only one animal was assessed per time point, so estimates of interanimal variability could not be determined. For the studies with radiolabeled isavuconazonium sulfate, we could not determine the contribution of the by-products of isavuconazole metabolism to the radioactivity in any tissues. Indeed, given that about half of the active isavuconazole moiety in humans is excreted as metabolites in urine (31), that was likely to be a substantial contributor to the radioactivity detected in kidney and urinary tract tissues. Furthermore, these studies were conducted in healthy animals, and so it is not clear whether the extent of tissue penetration might be substantially affected by inflammation associated with infection. Nevertheless, given the efficacy of isavuconazole in the range of tissues from the aforementioned preclinical studies, clinically relevant alterations in tissue penetration due to inflammation may be unlikely.

In conclusion, this study in rats suggests that there is wide distribution of isavuconazole into tissues, including into sanctuary sites, such as the brain, and that the tissue concentrations are sufficiently high to provide an antifungal effect. The study provides the experimental foundation for a further understanding of the broad clinical utility of isavuconazole.

## MATERIALS AND METHODS

### Study drugs.

The radiolabeled test article, [cyano-^14^C]isavuconazonium sulfate, was provided by Sekisui Medical Co., Ltd. The radiolabel was located on the active product of isavuconazonium sulfate (Fig. 1). The distribution of the unlabeled inactive moiety, BAL8728, was not monitored. Unlabeled isavuconazonium sulfate (25 mg/kg isavuconazole equivalent in 0.9% NaCl [10 ml/kg]) and voriconazole (50 mg/kg [10 ml/kg of a voriconazole {Vfend} oral suspension diluted 1/8 with 0.9% NaCl]) were used for the assessment of isavuconazole and voriconazole concentrations in brain tissue and plasma.

### Animals.

All animal procedures were performed in an Association for Assessment and Accreditation of Laboratory Animal Care (AAALAC)-accredited facility. The study was conducted in accordance with the Wisconsin Department of Health Services Radiation Protection Section (license no. 025-1076-01) and the FDA good laboratory practice (GLP) regulations in 21 CFR 58 (GLP for nonclinical laboratory studies), with the following exception: the radiolabeled test articles were not characterized in accordance with GLP or good manufacturing practice (GMP) regulations. Animal procedures and handling at Basilea Pharmaceutica International Ltd. complied with the accredited Bundesamt für Veterinärwesen (Basel, Switzerland) protocols and licenses. All animal procedures conducted at Covance were in compliance with Animal Welfare Act regulations (9 CFR 3).

Male Long-Evans rats (body weight range, 231 to 262 g) and male Sprague-Dawley rats (body weight range, 213 to 242 g) were sourced from Harlan Laboratories, Indianapolis, IN, USA. Male Wistar rats (body weight range, 297 to 352 g) were sourced from Harlan, The Netherlands. Animals were individually housed in suspended, stainless steel, wire mesh cages during acclimation and the test period. Environmental controls for the animal room were maintained at a temperature of 20 to 26°C, a relative humidity of 50% ± 20%, and a 12-h light/12-h dark cycle. Certified rodent diet number 2016C (Harlan) was provided *ad libitum*, except as specified in the dosing procedures. Water was provided *ad libitum*.

### QWBA studies of ^14^C-labeled isavuconazonium sulfate in pigmented rats following single-dose administration.

Ten male Long-Evans rats received [cyano-^14^C]isavuconazonium sulfate (5 mg/kg) orally in water. One animal per time point was sacrificed for QWBA. Animals under isoflurane anesthesia were sacrificed via exsanguination at 1, 2, 4, 8, 24, 72, 144, 336, and 672 h postdose. At termination, blood (2 to 10 ml) was collected in heparinized tubes and stored at 5°C until aliquoted for radioanalysis by LSC. The rats were immediately frozen solid in CO_2_–*n*-hexane for future analysis.

The quantitative distribution of ^14^C concentrations was assessed by QWBA using radioluminography (Covance Laboratories Inc., Madison, WI, USA). Forty-micrometer sections were collected on adhesive tape at six levels of interest in the sagittal plane using a Leica CM 3600 cryomicrotome and dried at −20°C (for a detailed description of cryosectioning for QWBA, see reference 32). All major tissues, organs, and biological fluids were examined. Gastrointestinal contents were not evaluated. Mounted sections were exposed on phosphorimaging screens, with blood standards being spiked with known levels of radioactivity for subsequent calibration of the image analysis software (for details on the linearity and variability of the standards, see references 33 and 34). Screens were exposed for 7 days and were scanned using whole-body autoradiography, and MCID software (Interfocus Imaging Ltd., Cambridge, UK) was used to quantitate the radioactivity. Autoradiographic standard image data were sampled using InterFocus Imaging Ltd. MCID analysis software to create a calibrated standard curve. Tissue concentrations were interpolated from each standard curve and converted from nanocuries per gram to microgram equivalents per gram on the basis of the test article specific activity (free base) after applying a correction factor of 1.1352 (sulfate/free form).

### QWBA study of ^14^C-labeled isavuconazonium sulfate in albino rats following repeated dose administration.

Seventeen male Sprague-Dawley rats received [cyano-^14^C]isavuconazonium sulfate (30 mg/kg/day orally in water; the dose was based on the salt form) for 21 days. Blood samples and bodies were collected for QWBA. Animals (one animal per time point) were sacrificed on day 1 at 1, 4, 8, and 24 h postdose, on days 7 and 14 at 1 h and 24 h postdose, and on day 21 predose (∼24 h postdose on day 20) and at 1, 4, 8, 24, 72, 168, 336, and 672 h postdose for QWBA. Collection of blood samples at termination, methods of sacrifice, carcass handling, and LSC and QWBA methods were those used in the single-dose study of the ^14^C-labeled isavuconazonium sulfate tissue distribution described above.

For both QWBA studies, the limit of quantification was 0.02 μg eq/g for ^14^C-labeled isavuconazonium sulfate in QWBA. Pharmacokinetic parameters (half-life [*t*_1/2_], AUC_inf_, and AUC from time zero to the last measurable time point [AUC_last_]) were calculated using WinNonlin software (professional edition, version 5.2; Certara USA, Inc., Princeton, NJ, USA).

### Liquid scintillation counting measurement of ^14^C-labeled isavuconazonium sulfate in blood and plasma.

For measurement of radioactivity in blood and plasma, blood samples (2 to 10 ml) from all animals used in the single- and repeated-dose experiments were collected in tubes containing sodium heparin and placed on ice. Duplicate weighed aliquots from each sample were taken to obtain measurements in plasma (prepared by centrifugation at 1,300 × *g* for 10 min at 5°C) or blood. For blood samples, PerkinElmer solubilizing agent was added to digest the samples (1 h, 60°C) before addition of 0.1 M disodium EDTA (to reduce foaming) and 30% hydrogen peroxide (to remove the color). Samples were stored overnight to allow the dissipation of foaming before analysis. Ultima Gold XR scintillation cocktail was added directly to plasma or digested blood, and the samples were shaken and analyzed by LSC.

### Brain and plasma levels of isavuconazonium, isavuconazole, and voriconazole in rats following a single oral dose of unlabeled isavuconazonium sulfate or voriconazole.

Two groups of 24 male Wistar rats received a single dose of either isavuconazonium sulfate (25 mg/kg [10 ml/kg] isavuconazole equivalent in 0.9% NaCl) or voriconazole (50 mg/kg [10 ml/kg of a voriconazole oral suspension diluted 1:8 with 0.9% NaCl]). The doses of isavuconazole and voriconazole were chosen to slightly exceed the clinically relevant human equivalent doses (approximately 20 mg/kg and 40 mg/kg, respectively). Blood samples were collected by cardiac puncture from three rats per time point (predose and 15 min, 30 min, and 1, 2, 3, 6, 8, and 24 h postdose) while the animals were under isoflurane anesthesia and placed in heparinized vials. The plasma proteins were precipitated by addition of a 5-fold excess of acetonitrile (containing 0.5% trifluoroacetic acid) for measurement of isavuconazonium, isavuconazole, and voriconazole concentrations using a validated liquid chromatography-tandem mass spectrometry (LC-MS/MS) method (see below). Immediately after terminal blood collection, the brains were collected, weighed, frozen in liquid nitrogen, and stored at −80°C until processing for assessment of the concentrations of isavuconazonium, isavuconazole, and voriconazole by LC-MS/MS. The rat brains were diluted 1:5 with water and homogenized for 60 s at a high velocity with an Ultra-Turrax tube disperser (IKA Werke GmbH & Co. KG, Staufen, Germany).

For LC-MS/MS, paraoxon and citric acid were first added to samples for analysis of isavuconazonium sulfate to prevent decomposition (final concentrations, 1 mM and 20 mM, respectively). Calibration and quality control (QC) solutions of isavuconazonium, isavuconazole, and voriconazole were prepared with 150 μl of an internal standard solution (10 ng/ml each of isavuconazonium-*d*_4_, isavuconazole-*d*_4_, and pyridooxazinone in acetonitrile-trifluoroacetate [TFA] at 100:0.05) and 50 μl of stock solutions of isavuconazonium, isavuconazole (in dimethyl sulfoxide [DMSO]), or voriconazole (in acetonitrile-TFA at 100:0.05) to the following final ranges of concentrations for calibration and QC: for isavuconazonium, 10 to 500 ng/ml and 25 to 250 ng/ml, respectively; for isavuconazole, 10 to 1,000 ng/ml and 25 to 2,500 ng/ml, respectively; and for voriconazole, 10 to 5,000 ng/ml and 25 to 2,500 ng/ml, respectively. Unknown rat plasma and brain homogenate samples (50 μl) were also mixed with 150 μl of the internal standard solution for analysis. All samples were centrifuged, supernatants were transferred to new tubes and diluted 10-fold if necessary (with plasma for plasma samples or with water for brain tissue samples), and 2 μl was injected into the LC-MS/MS instrument (Xevo TQ-S; Waters Corporation, Manchester, UK). The limit of quantification for all analytes was 10 ng/ml and 50 ng/g in plasma and brain, respectively.
